# Epidemiological characterization of COVID-19 in children under 18 years old in Mexico: an analysis of the pandemic

**DOI:** 10.3389/fped.2024.1440107

**Published:** 2025-01-07

**Authors:** Isamu Daniel Takane-Cabrera, Fanny Yasmin Ortega-Vargas, Ilen Adriana Díaz-Torres, Aldo Agustin Herrera-González, Antonio R. Villa, Miguel Leonardo García-León, Patricia Bautista-Carbajal, Miguel A. Pérez-Sastre, Luis Alberto Cortazar-Maldonado, Jorge Baruch Díaz-Ramírez, Rosa Maria Wong-Chew

**Affiliations:** ^1^ Infectious Diseases Research Laboratory, Research Division, Facultad de Medicina, Universidad Nacional Autónoma de México, Ciudad de México, Mexico; ^2^ Traveler’s Preventive Clinic, Research Division, Facultad de Medicina, Universidad Nacional Autónoma de México, Ciudad de México, Mexico

**Keywords:** COVID-19, SARS-CoV-2, children, Mexico, pandemic, incidence, waves, mortality

## Abstract

**Objective:**

The study aimed to describe the characteristics and risk factors associated with disease severity across six waves of COVID-19 in the pediatric population in Mexico.

**Methods:**

A cohort study was conducted using data from the Mexican Ministry of Health, covering the period from March 2020 to March 2023. The dataset included patients under 18 years of age with confirmed SARS-CoV-2 infection. Univariate, bivariate, and logistic regression analyses were performed to determine demographic and clinical characteristics, mortality across waves, and age group distributions.

**Results:**

Of the total cohort, 9.5% were children, with 497,428 confirmed cases. Among these, 50% were male, 4.4% required hospitalization, and there were 1,447 (0.03%) deaths. The highest prevalence was observed in the 12–17-year age group (52%), followed by the 5–11-year age group (32%), with incidence rates peaking towards the end of 2021 and the early 2022. Although the 0–2-year age group represented 9.6% of cases, it had higher hospitalization (40%), ICU admission (58%), and case fatality rate (CFR) (44%). Cardiovascular disease, hypertension, diabetes and immunosuppression were identified as risk factors for severe outcomes. The initial wave displayed the highest CFR (OR 5.28) especially in children aged 0–2 years.

**Conclusions:**

Children were less affected during the pandemic compared to adults; however, children under two years-old experienced more severe outcomes. Currently, with 95% of the population estimated to be immune due to vaccination and/or prior infection, children under 2 years of age are now at higher risk of severe disease and should be evaluated for vaccination as a public health policy.

## 1 Introduction

Globally, since the emergence of the SARS-CoV-2 (COVID-19) pandemic in China, early 2020 reports indicated that the adult population was disproportionately affected, with 98% of cases occurring in individuals over 18 years of age and only 2% in the pediatric population (1, 2).

Clinical manifestations of SARS-CoV2 infection in children vary, from asymptomatic cases to severe conditions such as pneumonia or Pediatric Inflammatory Multisystemic syndrome (PIMS) (3), which may require hospitalization and can lead to complications including death or long COVID-19 as a post-infectious syndrome. A systematic review of COVID-19 including patient series from China, Italy, Spain, and the United States reported that 5%–21% of cases in children were asymptomatic, while only 2% exhibited symptoms typical of upper respiratory tract infections (4, 5). Another systematic review found that 12% of infected children with SARS-CoV-2 showed symptoms, with a prevalence of 25% for cough, 9% for fatigue and 33% for fever, and 4% required intensive care (6).

In Mexico, from February 28, 2020, to March 31st, 2023, 497,428 confirmed cases (6.5%) (7) have been reported in individuals under 18 years old. Six waves of COVID-19 occurred, each associated with a distinct viral variant resulting from mutations in the genetic material of the virus, including Wuhan-HU1, B.1.1.159, Delta, Omicron, BA.4/BA.5 and XBB.1.5. These mutations affected the infectivity, pathogenicity, and immune evasion capabilities of the virus, contributing to outbreaks or more severe clinical presentations (8, 9).

The initial symptoms of COVID-19 documented in the pediatric population in Mexico included cough (53%), headache (53%), fever (47%), sore throat (33%), runny nose (29%), myalgia (28%), general discomfort (27%), arthralgia (23%), chills (21%), irritability (19%), diarrhea (17%), dyspnea (14%), chest pain (12%), abdominal pain (12%), conjunctivitis (10%), vomiting (7%), tachypnea (7%), and cyanosis (3%), with a case fatality rate CFR) of 1.3% (10).

The risk of infection was lower among children under 10 years of age and in school settings compared to adults, while adolescents in community and high schools' environments had a comparable risk (11).

Despite being the last age group to receive access to COVID-19 vaccination in Mexico (only children aged 5–17 years), the hospitalization and mortality rates in this population remained lower than those in adults (3.8 per 100,000 inhabitants). However, national data on the clinical characteristics and demographics of SARS-CoV-2 infection in children and adolescents are limited (12).

This study aimed to provide a descriptive analysis of the incidence, clinical and demographic characteristics, and risk factors associated with mortality in the pediatric population during the different COVID-19 waves in Mexico.

## 2 Materials and methods

### 2.1 National COVID-19 database

This study was conducted by analyzing a national database provided by the Ministry of Health (13), covering the period from March 2020 to March 2023. The dataset was filtered to focus on pediatric patients aged 18 years or younger with a probable diagnosis of SARS-CoV-2 infection. Variables associated with demographic characteristics and risk factors were extracted from the database, which recorded the presence of diabetes mellitus, hypertension, obesity, asthma, immunosuppression, tobacco exposure, cardiovascular diseases, chronic kidney disease, and chronic obstructive pulmonary diseases (COPD) as co-morbidities.

#### 2.1.1 Definition of co-morbidities included in the database

Diabetes was defined as a history of diabetes characterized by fasting plasma glucose >100 mg/dl or postprandial glucose <140 mg/dl (14). Hypertension was defined as a history of hypertension characterized by a blood pressure higher than 140/90 in two different time points (15). Obesity was defined by a body mass index of 30 kg/m^2^ or higher (16). Asthma was defined as a history of asthma characterized by wheezing, shortness of breath, coughing and chest tightness (17). Immunosuppression was defined as history of an impairment of the immune response resulting from conditions or factors intrinsic or extrinsic to the immune system because of malnutrition, metabolic disorders, use of immunosuppressive medications, chronic infections, malignancies, and severe trauma (18). Tobacco exposure was defined as history of an involuntary household exposure to tobacco smoke (19). Cardiovascular disease was defined as a history of cardiac impairment such as arrythmias, chronic cardiac failure, ischemic cardiomyopathy, among others (20). Chronic kidney disease was defined as a history of chronic kidney disease characterized by glomerular filtration rate of less than 60 ml/min per 1.73 m^2^, or markers of kidney damage, or both, of at least 3 months duration, regardless of the underlying cause (21). Chronic obstructive pulmonary disease (COPD) was defined as a history of COPD characterized by persistent airflow limitation that is usually progressive and associated with an enhanced chronic inflammatory response in the airways and the lung to noxious particles or gases (22).

#### 2.1.2 Subjects included in the analysis

**Figure 1:**
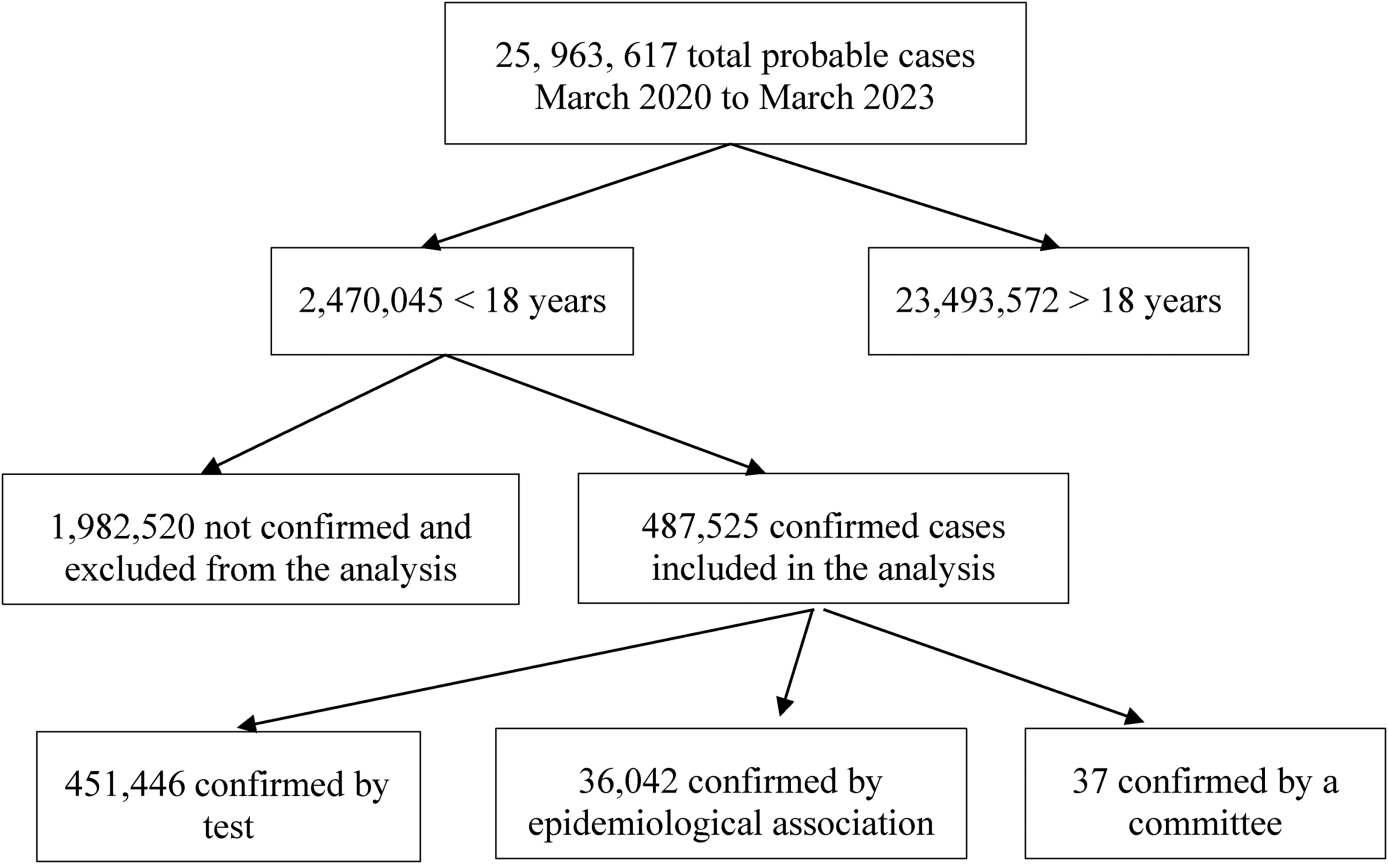
Flowchart of patients included in the study.

Initially, a COVID-19 database comprising 25,963,617 individuals was filtered, resulting in a subset of 2,470,045 patients younger than 18 years old. This subset was further filtered to include only those with SARS-CoV-2 infection (Figure 1). A confirmed case was defined as individuals with a positive polymerase chain reaction test (PCR) for SARS-CoV-2, a positive antigen tests, or a positive result determined through clinical presentation and epidemiological association, or confirmation by a review committee (23, 24). During the pandemic, PCR or antigen testing for SARS-CoV-2 was not widely available throughout the country, especially at the onset. Consequently, the government classified patients as COVID-19 cases based on suggestive symptoms and known contact with a confirmed case (epidemiological contact), or in deceased cases, on clinical history assessed by a review committee, even in the absence of a test (23, 24).

### 2.2 Criteria for variables definition

Patients were grouped by age into four categories: 0–2 years, 3–4 years, 5–11 years, and 12–17 years. This classification was based on the national vaccination strategy, which prioritized the 12–17 age group followed by the 5–11 age group. Furthermore, the highest mortality rates had previously been observed in infants aged 0–2 years (10).

The clinical severity of the disease was categorized into three levels: those who developed pneumonia, those who required intubation, and those who died, according to the database definitions.

Suspected Case: An individual of any age who, within the past 7 days, has presented with two or more signs and symptoms (such as cough, fever, or headache), accompanied by at least one of the following symptoms (dyspnea as a severity indicator, arthralgia, myalgia, sore throat, rhinorrhea, conjunctivitis, or chest pain).

Severe Acute Respiratory Infection Case: Any individual who meets the criteria for a suspected case of mild respiratory illness and additionally presents with difficulty breathing and is hospitalized.

Confirmed Case: An individual who meets the operational definition of a suspected case and has a laboratory-confirmed PCR diagnosis, a positive antigen tests, or a positive result determined through epidemiological association, clinical presentation, or confirmation by a review committee.

Pneumonia: patients who were clinically diagnosed with pneumonia.

Intubation: patients who required mechanical ventilation and were intubated (13, 23).

### 2.3 Definition of COVID-19 waves during the pandemic in Mexico

An additional classification stratified patients according to the COVID-19 pandemic waves, considering the sample collection and/or medical appointment dates. This stratification was aligned with national epidemiological reports to ensure accurate incidence reporting.

**Figure 2:**
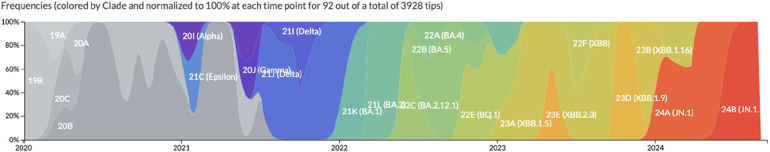
COVID-19 variants in Mexico. Downloaded from https://nextstrain.org/ncov/gisaid/global/all-time?f_country=Mexico under the The CC-BY-4.0 (accessed on September, 2024).

An open database from the General Directorate of Epidemiology of the Ministry of Health was utilized to delineate the timeline of each wave. The intervals between each wave were adjusted to facilitate objective analysis, with each wave linked to the predominant circulating variant, based on data from GISAID and the COVIGEN (acronym for the Spanish name, Mexican Consortium of Genomic Vigilance) (25). The following wave periods were defined: Wave 1 (Wuhan-HU1) from March 1st, 2020, to September 23rd, 2020; Wave 2 (B.1.1.519) from September 24th, 2020, to May 15th, 2021; Wave 3 (Delta variant) from May 16th, 2021, to December 15th, 2021; Wave 4 (Omicron variant) from December 16th, 2021, to May 15th, 2022; Wave 5 (BA.4 and BA.5 variants) from May 16th, 2022, to October 15th, 2022; and Wave 6 (XBB 1.5 variant) from October 16th, 2022, to March 6th, 2023 (Figure 2).

### 2.4 Statistical analysis

Descriptive and bivariate statistics were used to analyze the variables, with percentages also calculated. Analyses were performed using the Statistical Package for Social Sciences IBM® software (SPSS version 25). Categorical variables were compared using the *χ*^2^ test or Fisher's exact test, as appropriate, while continuous variables were compared using the student's *t*-test. A *p*-value <0.05 was considered statistically significant. Logistic regression was conducted to assess lethality across different waves. The case fatality rate of each wave was compared to the wave with the lowest lethality.

Additionally, an analysis of comorbidities associated with SARS-CoV-2 case fatality rate was performed for each wave.

COVID-19 incidence was calculated by dividing the number of positive cases (diagnosed through testing and/or clinical criteria) per semester by the total population of children in each age group, then multiplying by 100,000. Lethality was calculated similarly, using the number of deaths.

### 2.5 Ethical considerations

The study was approved by the Research and Ethics Committees of the Faculty of Medicine, Universidad Nacional Autónoma de México (FM/DI/093/2020). Informed consent was not required because the work was done with a secondary database analysis where the identity of the subjects was not available.

## 3 Results

### 3.1 COVID-19 confirmed cases included in the analysis

The COVID-19 case registry from the Mexican Ministry of Health, covering the period from March 2020 to March 2023, included a total of 25,963,617 individuals. Among these, 2,470,045 were children under 18 years of age (9.5%), with 487,525 children having a clinical and/or laboratory-confirmed COVID-19 diagnosis and included in the analysis (Figure 1). Of the confirmed cases, 451,446 (92.6%) were verified by a diagnostic test, with 113,298 (22.7%) confirmed by PCR and 374,227 (77.3%) by antigen testing. An additional 36,042 cases (7.4%) were identified based on clinical symptoms and epidemiological association (contact with a confirmed test-positive case), while 37 cases (0.000076%) were confirmed by a committee.

### 3.2 Incidence according to different variables

**Figure 3:**
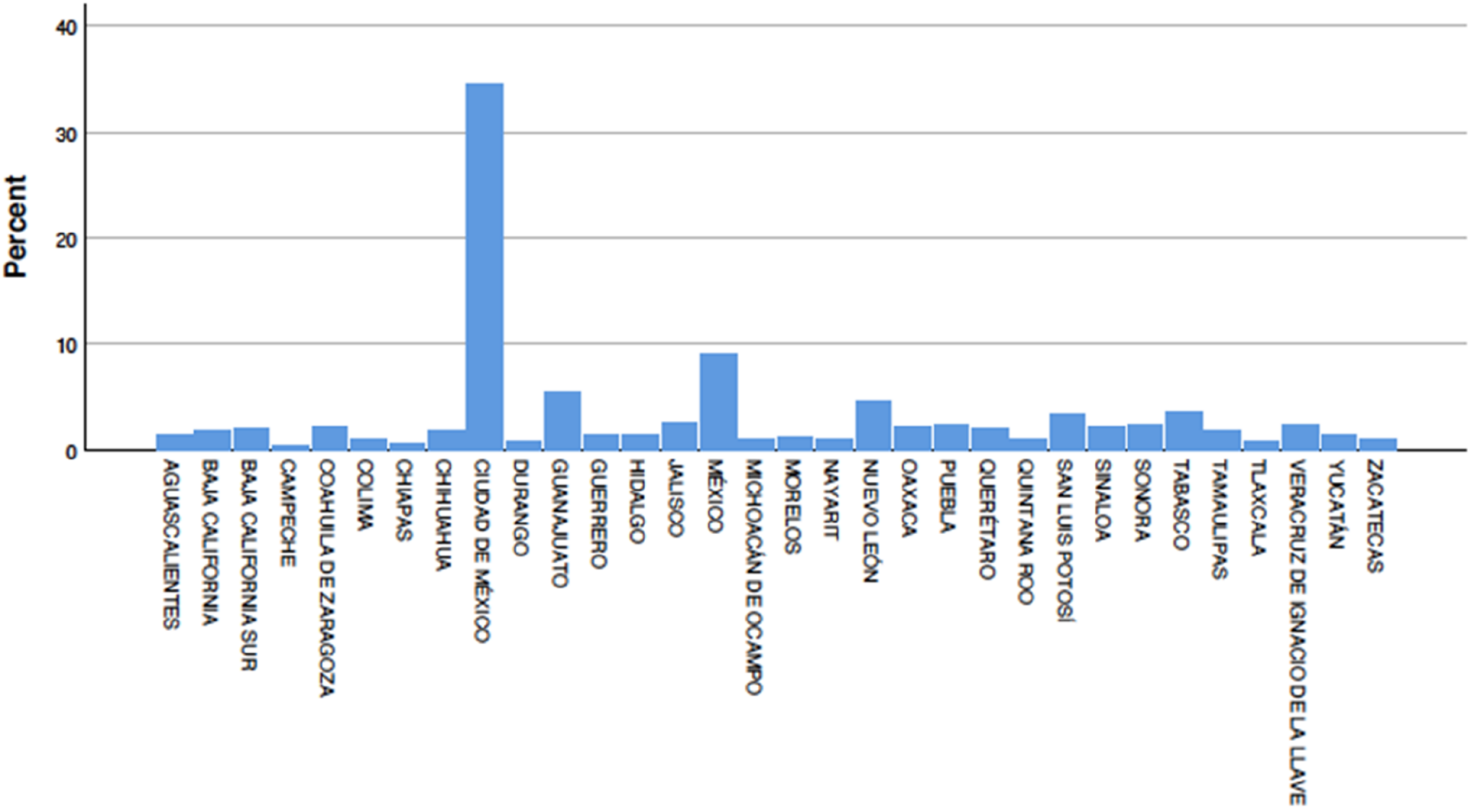
Distribution of pediatric COVID-19 cases by state.

The states most affected, with the highest proportion of pediatric cases, were Mexico City (34.5%), Mexico state (9.2%), Guanajuato (5.6%), Nuevo León (4.5%), Tabasco (3.5%), San Luis Potosí (3.4%), with the remaining states each less than 2% of cases (Figure 3).

Of this population, 246,855 (50.6%) were male and 240,670 (49.4%) were female.

**Figure 4:**
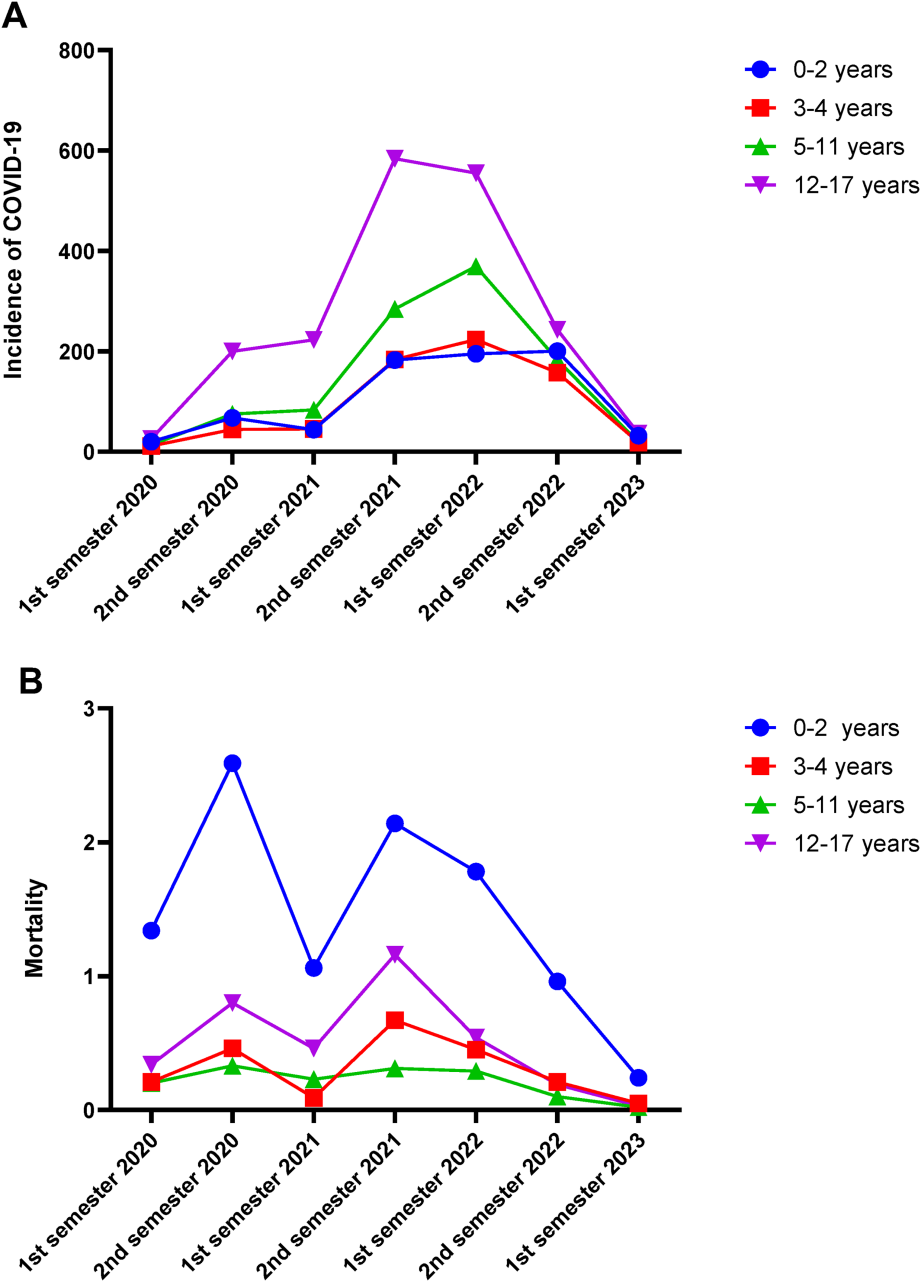
Incidence of COVID-19 cases and deaths in Mexican children during the SARS-CoV2 pandemic. The figure shows the semiannual distribution of the incidence **(A)** and mortality rate **(B)** of SARS-CoV-2 cases per 100,000 children under 18 years of age, from 2020 to 2023, categorized by age groups in Mexican children with COVID-19. The first semester of 2023 includes only from January to March 2023. The incidence and mortality rate are cases per 100,000 children.

Based on age classification, the group with the highest prevalence was those aged 12–17 years, accounting for 52.1% of cases, followed by the 5–11-year-old group with 32.4% cases, the 0–2-year-old group with 9.6%, and the 3–4-year-old group with 5.9% (Table 1). The highest incidence rate for the 12–17-year-old group was observed in the second half of 2021, with an incidence of 584.01 per 100,000 people. In contrast, incidence rates for the 3–4 year and 5–11year groups were 223.35 and 369.22 per 100,000 people, respectively, peaking in the first half of 2022. The peak incidence in the 0–2-year-old group, at 200.3 per 100,000 people, was the last to occur, reaching its maximum in the second half of 2022 (Figure 4A).

**Table 1:** Demographic characteristics of children with confirmed COVID-19 diagnosis.

Characteristics	0–2 years *n* (%)	3–4 years *n* (%)	5–11 years *n* (%)	12–17 years *n* (%)	Total *n* (%)	*p*
Confirmed cases	46,715 (9.6)	28,877 (5.90)	157,970 (32.40)	253,963 (52.10)	*n* = 487,525	
Country region						
North	10,858 (23.2)	5,818 (20.1)	28,529 (18.1)	49,050 (19.3)	94,255 (19.3)	<0.001
% age/North	11.50	6.20	30.30	52.00		
Center	18,632 (39.9)	13,340 (46.2)	81,327 (51.5)	127,530 (50.2)	240,829 (49.4)	
% age/Center	7.70	5.50	33.80	53.00		
West	10,881 (23.3)	6,241 (21.6)	28,871 (18.3)	45,244 (17.8)	91,237 (18.7)	
% age/West	11.90	6.80	31.60	49.60		
South	6,344 (13.6)	3,478 (12.0)	19,243 (12.2)	32,139 (12.7)	61,204 (12.6)	
% age/South	10.40	5.70	31.40	52.50		
Gender						
Female	21,575 (46.2)	13,463 (46.6)	75,743 (47.9)	129,889 (51.1)	240,670 (49.4)	<0.001
% female/age	9.00	5.60	31.50	54.00		
Male	25,140 (53.8)	15,414 (53.4)	82,227 (52.1)	124,074 (48.9)	246,855 (50.6)	
% age/male	10.20	6.20	33.30	50.30		
Hospital area						
Ambulatory	38,181 (81.7)	26,880 (93.1)	152,856 (96.8)	248,298 (97.8)	466,215 (95.6)	<0.001
% age/ambulatory	8.20	5.80	32.80	53.30		
Hospitalization	8,534 (18.3))	1,997 (6.9)	5,114 (3.2)	5,665 (2.2)	21,310 (4.4)	
% age/hospitalization	40.00	9.40	24.00	26.60		
ICU	971 (2.1)	89 (0.3)	244 (0.2)	372 (0.1)	1,676 (0.3)	<0.001
% age/IC	57.9	5.3	14.6	22.2		
Indigenous	465 (15.2)	173 (5.6)	823 (26.9)	1,601 (52.3)	3,062 (100)	

The vertical columns represent the number and percentage of children in each age group for the corresponding characteristic. The percentages within each characteristic (horizontal rows) represent the proportion of children in the different age groups who possess that characteristic.

A *p*-value of <0.05 is considered statistically significant.

**Figure 5:**
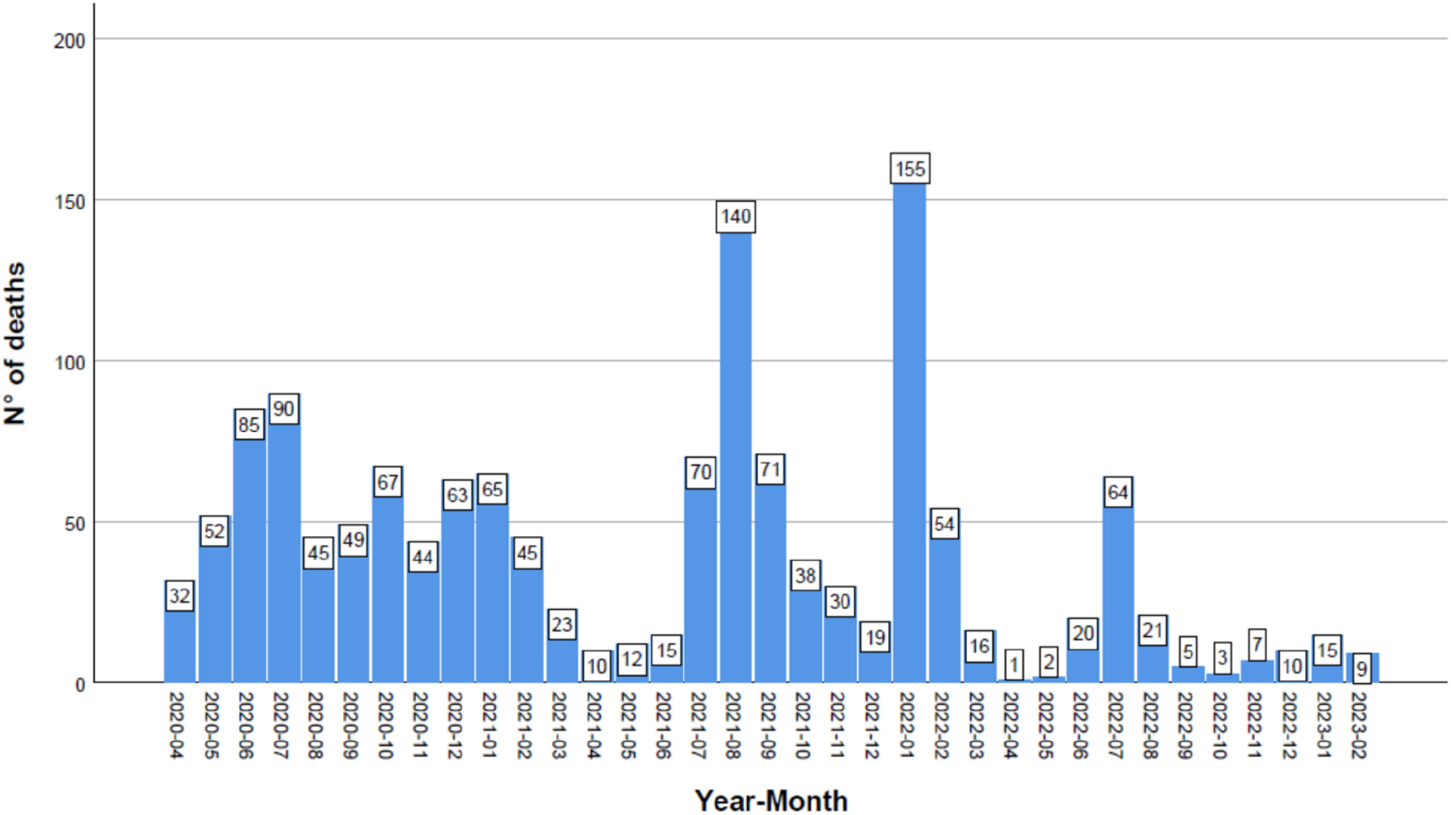
Number of deaths in the pediatric population in Mexico during the COVID-19 pandemic. The graph illustrates the number of deaths per month and per year, with the highest monthly peaks occurring during the predominance of the Delta and Omicron waves.

Contrary to incidence patterns, mortality rates were higher in the 0–2-year-old group, particularly during the second half of 2020, which aligns with the circulation of the B.1.1.519 variant, and in the second half of 2021, coinciding with the Omicron variantś prevalence. Mortality peaked again in the first half of 2022 with the circulation of BA.4/BA.5 variants (Figure 4B). There were two peaks in monthly death counts: January 2022 and August 2021, corresponding to the circulation of the Omicron and Delta variants, respectively (Figure 5).

There were 3,062 (0.7%) indigenous children with COVID-19, with 1,510 (49%) female and 1,552 (50.7%) males, the highest proportion being in the 12–17 years age group with 1,601 individuals (52.3%), followed by the 5–11 years group with 823 (26.9%), the 0–2 years group with 465 (15.2%), and the 3–4 years group with 173 (5.6%) (*p* < 0.001) (Table 1). Geographically, from the indigenous children 443 (14.5%) were from the North region, 720 (23.5%) from the Center, 582 (19%) from the West, and 1,316 (43%) from the South.

Geographically, most cases were concentrated in the central region of the country (49.4%). Of the total cases, 466,215 patients (95.6%) were ambulatory, while 21,310 (4.4%) required hospitalization. Notably, the 0–2 years age group exhibited the highest hospitalization rate, accounting for 8,534 cases (18.3%), and 2% of ICU admissions (representing 58% of all admissions across age groups) (Table 1).

### 3.3 Risk factors

Among patients with SARS-CoV-2 infection, asthma and obesity were the most prevalent comorbidities, accounting for 2.4% and 2.2% cases, respectively. Immunodeficiency was the third most common risk factor, present in 0.5% of cases, with the 0–2-year and 3–4-year groups showing the highest rates (both 0.7%). Tobacco smoke was notably high in the 12–17 age group, with 1,039 cases (0.4%), and obesity was most common in this group as well, accounting for 3% (70% of cases across all age groups) (Table 2).

**Table 2:** Risk factors.

Risk factors	0–2 years *n* = 46,715 *n* (%)	3–4 years *n* = 28,877 *n* (%)	5–11 years *n* = 157,970 *n* (%)	12–17 years *n* = 253,963 *n* (%)	Total *n* = 487,525 *n* (%)	*p*
Exposure to tobacco smoke						
Yes	146 (0.3)	31 (0.1)	185 (0.1)	1,039 (0.4)	1,401 (0.3)	<0.001
% in each age group	10.40	2.20	13.20	74.20		
Comorbidities						
Asthma	256 (0.5)	415 (1.4)	3,922 (2.5)	6,889 (2.7)	11,482 (2.4)	<0.001
% age/asthma	2.20	3.60	34.20	60		
Obesity	398 (0.9)	129 (0.4)	2,613 (1.7)	7,494 (3.0)	10,634	<0.001
% age/obesity	3.70	1.20	24.60	70.50	2.2	
Immunosuppression	327 (0.7)	201 (0.7)	874 (0.6)	947 (0.4)	2,349 (0.5)	<0.001
% age/immunosuppression	13.90	8.60	37.20	40.30		
Diabetes	258 (0.6)	63 (0.2)	391 (0.2)	1,079 (0.4)	1,791 (0.4)	<0.001
% age/diabetes	14.40	3.50	21.80	60.20		
Cardiovascular disease	469 (0.1)	111 (0.4)	470 (0.3)	721 (0.3)	1,771 (0.4)	<0.001
% age/CVD	26.50	6.30	26.50	40.70		
Hypertension	333 (0.7)	60 (0.2)	276 (0.2)	748 (0.3)	1,417 (0.3)	<0.001
% age/hypertension	23.50	4.20	19.5	52.80		
Chronic Kidney Disease	69 (0.1)	32 (0.1)	226 (0.1)	529 (0.2)	856 (0.2)	<0.001
% age/CKD	8.10	3.70	26.40	61.80		
COPD	56 (0.1)	16 (0.1)	85 (0.1)	176 (0.1)	333 (0.1)	<0.001
% age/COPD	16.80	4.80	25.50	52.90		

The vertical columns represent the number and percentage of children in each age group with the corresponding risk factor. The percentages within each characteristic (horizontal rows) represent the proportion of children across different age groups who exhibit that risk factor.

A *p*-value of <0.05 is considered statistically significant.

Disease severity was classified into two groups: patients who developed pneumonia (9,916 cases, 2.0%) and those who required intubation (1,294 cases, 0.3%). The highest proportion of pneumonia and intubation cases occurred in the 0–2-year age group, representing 7.2% (3,333 cases) and 1.4% (677 cases) of all cases, respectively. A total of 1,447 deaths (0.03%) were recorded, with the 0–2 age group accounting for the highest proportion of deaths (44.8% across age groups, 1.4% of all the cohort), followed by the 12–17 age group with 33.1% of deaths across ages (0.2% of all the cohort) (Table 3).

**Table 3:** Clinical status of severity*.*

Clinical Status	0–2 years *n* = 46,715 *n* (%)	3–4 years *n* = 28,877 *n* (%)	5–11 years *n* = 157,970 *n* (%)	12–17 years *n* = 253,963 *n* (%)	Total *n* = 487,525 *n* (%)
Condition					
Pneumonia	3,333 (7.2)	739 (2.6)	2,283 (1.5)	3,561 (1.4)	9,916 (2.0)
Intubation	677 (1.4)	80 (0.3)	219 (0.1)	318 (0.1)	1,294 (0.3)
Clinical outcome					
Death	648 (1.4)	92 (0.3)	228 (0.1)	479 (0.2)	1,447 (0.3)
% age/death	44.80	6.40	15.80	33.10	100

This table presents the clinical status of severe COVID-19 among each age group. The “*n*” and “%” represent the number and percentage of the total sample in each age group. The “% age/death” corresponds to the percentage of deaths within each age group.

A comprehensive analysis of the disease severity across SARS-CoV-2 variants revealed that the highest percentage of deaths occurred during the first wave (Wuhan-HU1), especially in children aged 0–2 years (164 cases, 0.7%), followed by the 12–17-year age group (99 cases, 0.42%). Throughout the COVID-19 pandemic, the CFR decreased consistently across all age groups, except during the XBB.1.5 wave, which exhibited a slight increase, especially among the 0–2 age group (tripling the lethality observed during BA.4/BA.5). The lethality rate was significantly higher in males than females. Geographically, the central region recorded the highest number of deaths across all variant's waves (Table 4).

**Table 4:** Prevalence of lethality by age, sex and region during the different waves.

Predominant variant	Wuhan-HU1 *n* = 23,274 *n* (%)	B.1.1.519 *n* = 67,495 *n* (%)	Delta *n* = 144,308 *n* (%)	Omicron *n* = 123,262 (%)	BA.4 BA.5 *n* = 111,458 *n* (%)	XBB 1.5 *n* = 17,728 *n* (%)	*p*
Age							
0–2 years	164 (0.7)	157 (0.2)	138 (0.09)	104 (0.08)	58 (0.05)	27 (0.15)	<0.001
3–4 years	21 (0.09)	13 (0.01)	27 (0.01)	18 (0.014)	10 (0.008)	3 (0.05)	<0.001
5–11 years	62 (0.2)	62 (0.09)	49 (0.03)	45 (0.03)	16 (0.014)	5 (0.02)	<0.001
12–17 years	99 (0.42)	99 (0.14)	161 (0.11)	72 (0.05)	28 (0.02)	9 (0.05)	<0.001
Sex							
Female	159 (0.68)	146 (0.21)	186 (0.12)	109 (0.08)	65 (0.05)	19 (0.1)	<0.001
Male	187 (0.8)	185 (0.27)	189 (0.13)	130 (0.1)	47 (0.04)	25 (0.14)	<0.001
Region							
North	82 (0.35)	90 (0.13)	94 (0.06)	60 (0.0.04)	21 (0.01)	5 (0.02)	<0.001
Center	121 (0.51)	126 (0.33)	114 (0.07)	67 (0.05)	33 (0.02)	13 (0.07)	<0.001
West	55 (0.23)	74 (0.1)	73 (0.05)	65 (0.05)	30 (0.02)	16 (0.09)	<0.001
South	88 (2.4)	41 (0.06)	94 (0.06)	47 (0.03)	28 (0.02)	10 (0.05)	<0.001

The number and proportion of deaths in each age, sex, and region group across the different waves of the pandemic were calculated. A *p*-value of <0.05 was considered statistically significant.

The results indicated that cardiovascular disease (OR: 2.56) was the strongest predictor of mortality, followed by hypertension (OR: 1.99), diabetes (OR: 1.60), and immunosuppression (OR: 1.57) particularly during the first wave. Notably, asthma appeared to be a protective factor (Table 5).

**Table 5:** Risk factors for lethality associated with different waves of the pandemic.

Risk factors	Wuhan-HU1 *n* (%)	B.1.1.519 *n* (%)	DELTA *n* (%)	OMICRON *n* (%)	BA.4/BA.5 *n* (%)	XBB 1.5 *n* (%)	*p*	95% CI	OR
Domestic smoking	3 (1.6)	4 (0.9)	0 (0.0)	0 (0.0)	0 (0.0)	0 (0.0)	0.094	(0.216–1.255)	0.521
Diabetes	17 (10.1)	23 (6.1)	15 (3)	5 (1.4)	0 (0)	1 (1.4)	<0.001	(1.157–2.216)	1.602*
COPD	0 (0.0)	3 (4.3)	0 (0.0)	1 (1.3)	0 (0.0)	1 (9.1)	0.056	–	–
Asthma	4 (0.5)	8 (0.4)	4 (0.1)	2 (0.1)	2 (0.1)	0 (0)	0.007	(0.277–0.714)	0.445*
Immunosuppression	41 (9.6)	28 (7.9)	27 (7.0)	22 (4.0)	14 (2.9)	6 (4.2)	<0.001	(1.285–1.920)	1.571*
Hypertension	14 (9.6)	21 (6.9)	19 (5.1)	4 (1.4)	1 (0.4)	0 (0.0)	<0.001	(1.407–2.826)	1.994*
Cardiovascular diseases	22 (10.7)	23 (7.3)	26 (6.8)	20 (4.7)	5 (1.5)	4 (3.9)	<0.001	(2.007–3.284)	2.567*
Obesity	23 (2.3)	26 (1.2)	41 (1.2)	3 (0.1)	3 (0.2)	2 (0.8)	<0.001	(1.097–1.793)	1.402*
Chronic kidney disease	14 (12.8)	18 (11.5)	13 (6.3)	10 (5.3)	3 (1.8)	2 (5.9)	0.003	(1.908–3.715)	2.663*
Intubated	169 (46.2)	115 (45.8)	125 (45.8)	78 (42.2)	51 (37.8)	21 (25.0)	0.007	(0.985–1.006)	0.995

The number and percentage of each risk factor were calculated from the total sample for each wave. COPD, Chronic obstructive pulmonary disease; OR, odds ratio; CI, confidence Interval.

* *p*-value <0.05 is considered statistically significant.

Significant differences were observed across waves, with the first wave showing the highest lethality (OR: 5.28), which subsequently decreased over time (Table 6).

**Table 6:** Lethality of the different waves of the COVID-19 pandemic in Mexico.

	OR	95% CI	*p*
Wuhan-HU1	5.285	(3.799–7.354)	<0.001
B.1.1.519	4.802	(3.449–6.686)	<0.001
Delta	3.176	(2.289–4.406)	<0.001
Omicron	2.438	(1.744–3.407)	<0.001
BA.4/BA.5	1.373	(0.957–1.971)	0.086

Logistic regression analysis results for lethality among different waves compared to the sixth wave (XBB.1.5). OR, odds ratio; CI, confidence interval.

*p*-value <0.05 statistically significant.

In this study, indigenous population was used as a proxy for low socioeconomic status. Indigenous status was identified as a statistically significant risk factor for pneumonia (OR: 3.2, 95% CI: 2.7–3.7), intubation (OR: 6.2, 95% CI: 4.6–8.2), ICU admission (OR: 4.6, 95% CI: 3.4–6.1), and death (OR: 4.3, 95% CI: 3.1–5.9).

## 4 Discussion

This study delineated the incidence, demographic, and clinical characteristics of COVID-19 among children in Mexico, while identifying and evaluating risk factors associated with disease lethality, thereby providing a comprehensive understanding of COVID-19´s the progression in the pediatric population during the pandemic.

Although COVID-19 was initially reported to primarily impact the adult population, children have also been affected. However, due to the lower proportion of pediatric cases, children have been considered at reduced risk for severe outcomes and fatalities, resulting in limited studies on this age group (26). In countries such as the United States, the United Kingdom, and China, a higher hospitalization rate has been noted among children aged 1–4 years (27). In alignment with these findings, over 95% of cases in this study were managed on an outpatient basis, with only 0.3% requiring intensive care and 4.4% requiring hospitalization. Among hospitalized cases, 40% were children aged 0–2 years, followed by the 3–4-year-old group, confirming that while pediatric cases are typically mild or asymptomatic, disease severity may increase in early infancy.

While children under 4 years are particularly vulnerable to severe outcomes, other age groups remain relevant. Throughout the pandemic, the highest number of cases were among adolescents aged 12–17 years. However, despite the lower number of cases, the 0–2-year age group had the highest percentage of recorded deaths (44.8% across age groups), consistent with findings from Most et al. (28).

This discrepancy may be attributed to two factors: the immune system immaturity in very young patients, which may increase their susceptibility to severe diseases and the presence of chronic diseases and other risk factors among older patients, which may contribute to disease progression and fatal outcomes.

In this study, cardiovascular diseases, hypertension, diabetes, immunosuppression, and obesity emerged as primary risk factors associated with lethality. A cross-sectional analysis involving 43,465 patients aged 18 or younger with COVID-19 similarly identified type 1 diabetes, congenital heart and cardiovascular anomalies, obesity, hypertension, epilepsy, neuropsychiatric disorders, and chronic diseases, as risk factors for hospitalization or severe disease (29).

Asthma emerged as notable condition in our study. Initially, the Mexican Clinical Guide for the COVID-19 treatment identified asthma as a risk factor for severe COVID-19 in both children and adults (30), based on the heightened susceptibility of asthma patients to viral respiratory infections (31).

However, subsequent studies in Mexico and other countries did not find a significant association between asthma and hospital admission (32), suggesting that asthma might serve as a protective factor. Indeed, allergic asthma (33), has been associated with lower severity and reduced ACE2 receptor expression (29), which is crucial for the virus's entry into the host cell (34). Our study aligns with these findings, identifying asthma as a protective factor.

From a Public Health perspective, the pandemic underscored social and economic disparities in Mexico (35, 36), including social inequitable healthcare access. In Mexico, states with high marginalization indices are primarily located in the southern region (37). This study included fewer participants from these areas than from other regions yet observed higher lethality during the first wave (2.4% vs. 0.35% in the North, 0.51% in the Center, and 0.23% in the West). In this study, indigenous population served as a proxy for low socioeconomic status and was identified as a risk factor for pneumonia, intubation, and death. The highest proportion of the indigenous population was in the South, which is consistent with the region's highest mortality and lowest reporting rates. This trend is consistent with many authors highlighting the association between poverty and a worse disease outcome (38, 39). In this context, it is possible that the lower number of reported cases was due to the limited access to diagnosis and health services in these marginalized regions. Another observation is that most cases are concentrated in the central region of the country. This is due to Mexico City's large population, which, along with its metropolitan area, totals approximately 22,281,442 people (18% of the total population). Consequently, a high number of COVID-19 cases were recorded in this central region. Additionally, greater access to healthcare services and higher testing rates contributed to the increased case detection in this area.

Lastly, this study examined the lethality of different COVID-19 variants over time in Mexico, revealing significant differences in lethality across pandemic waves and in the demographic characteristics of deceased patients. Other studies in Mexico have noted that the frequency of pediatric cases of COVID-19 in different waves correlates with school closures and social distancing measures.

We observed that the first two waves exhibited higher lethality and lower incidence, whereas subsequent waves showed increased incidence in the pediatric population due to the transition from confinement to schools reopening in the third and fourth waves (36). Additionally, lower lethality was observed as new variants of SARS-CoV-2 emerged, consistent with our findings. This shift may reflect the progressive increase in vaccination coverage during the second and third waves, the development of natural immunity in the population, and the establishment of hybrid and herd immunity (38).

One study limitation was the use of a secondary database, which included significant underreporting. Nevertheless, this dataset represents the only available national information.

During the pandemic, adults, especially older adults, were prioritized for vaccination due to higher severe disease risk (39). However, as 95% of the population now has immunity through vaccination and/or infection (40), infants under 2 years are increasingly susceptible and more prone to severe disease and mortality and may benefit from vaccination as a public policy measure.

## 5 Conclusion

This study evaluated the impact of the COVID-19 pandemic on the pediatric population in Mexico, with the highest lethality and risk observed in children under two years. Despite an overall increase in cases over time, a decrease in severe cases and fatalities was observed, likely due to natural immunity and adult vaccination, both of which have altered the disease trajectory. However, chronic diseases remain critical; cardiovascular diseases, hypertension, and diabetes were the primary risk factors, with the greatest lethality peaking during the first wave, which was associated with the Wuhan-HU1 variant. Prevention strategies targeting susceptible individuals, particularly children without immunity to SARS-CoV2, should be considered in public policy through vaccination efforts.

## Data Availability

The original contributions presented in the study are included in the article/Supplementary Material, further inquiries can be directed to the corresponding author.
